# Psychosomatic Symptoms in Terminally Ill Cancer Patients and Its Relation With Using Complementary and Alternative Medicines: A Cross-Sectional Study in Southeast Iran

**DOI:** 10.3389/fpsyt.2022.871917

**Published:** 2022-05-17

**Authors:** Mahlagha Dehghan, Fatemeh Sadat Hoseini, Mohammad Ali Zakeri

**Affiliations:** ^1^Nursing Research Center, Kerman University of Medical Sciences, Kerman, Iran; ^2^School of Nursing and Midwifery, Kerman University of Medical Sciences, Kerman, Iran; ^3^Non-Communicable Diseases Research Center, Rafsanjan University of Medical Sciences, Rafsanjan, Iran; ^4^Social Determinants of Health Research Centre, Rafsanjan University of Medical Sciences, Rafsanjan, Iran

**Keywords:** alternative and complementary medicines, physical symptoms, psychological symptoms, quality of life, cancer

## Abstract

**Background:**

Cancer patients face various problems and complications, which they address through various complementary and alternative medicines (CAM). The aim of this study was to investigate the relationship between CAM and psychosomatic symptoms in terminally ill cancer patients.

**Methods:**

This cross-sectional study was performed on 221 terminally ill cancer patients (based on metastatic stage and according to the physicin diagnosis) in southeastern Iran. Convenience sampling was used to select terminally ill cancer patients. Using questionnaires like the demographic and clinical information questionnaire, Edmonton Symptom Assessment Scale (ESAS), Hospital Anxiety and Depression Scale (HADS), CAM questionnaire and satisfaction with the use of CAM, the researcher was able to compile a comprehensive picture of the population.

**Results:**

The mean age of the participants was 51.66 ± 13.34 years. The majority of the samples were female, married, educated, and unemployed. The mean score for the physical symptoms of the participants according to ESAS was 22.25 ± 17.57 which was less than the midpoint of the scale (the possible score of ESAS was 0–100). Only 2.7% (*n* = 6) and 0.9% (*n* = 2) of the participants had mild and moderate anxiety, respectively, and the other participants' anxiety levels were normal. Only 7.7% (*n* = 17) and 4.1% (*n* = 9) of the participants had mild and moderate depression, respectively, and the other participants' depression levels were normal. Last year, 87.3% of the participants used at least one type of CAM. Aside from prayer, 42.1% of the participants used at least one type of CAM in the last year. Prayer was used by 83.7% of the participants, medicinal plants by 35.8%, massage by 9.5%, dietary supplements by 3.6%, wet cupping by 3.2%, relaxation and meditation by 2.7%, dry cupping by 2.4%, and acupuncture by 0.5%. The common reason for using CAM was to reduce the stress and anxiety caused by cancer and to treat it. There were no significant differences in physical and psychological symptoms between the CAM-users and non-CAM users.

**Conclusion:**

Patients with cancer have a relatively low level of psychosomatic symptoms, and the primary reason for using CAM was to relieve stress and anxiety associated with cancer and treat it. However, psychosomatic symptoms were the same for CAM and non-CAM users. Because so many people with cancer use CAM, future studies should look into why and how CAM is used.

## Introduction

There have been numerous attempts in the last few decades to prevent and control diseases, but despite these efforts, chronic disease rates continue to rise (1). In medicine, cancer refers to a broad range of diseases that can affect any part of the body. Cancer is the main cause of mortality throughout the world, and ~10 million people died from cancer worldwide in 2020 (2). Palliative care is available to treat and relieve cancer symptoms as well as improve the quality of life of terminally ill cancer patients. In this regard, effective public health strategies, such as social and home care, are required to provide pain relief and palliative care for cancer patients (2).

Due to negative thoughts and worries about the disease, patients with cancer experience a high level of psychological stress, anxiety, and incompatibility (3). However, these patients' anxiety is exacerbated by persistent physical symptoms associated with treatment, such as pain, fatigue, and psychosis, caused by fear of recurrence and changes in family relationships (4). Anxiety is a negative emotion that occurs in response to a perceived risk, which can be real or imagined, and can come from an external or internal source (5). However, some crises, such as the COVID-19 outbreak, can enhance this anxiety (6, 7).

Studies have shown that the coronavirus epidemic poses serious problems for cancer patients who may be at high risk of death if they become infected (8–11). Cancer patients infected with COVID-19 may present with unusual symptoms (12). Patients with cancer had a higher rate of mechanical ventilation and COVID-19-induced mortality (11). Also, they are at significantly greater risk of COVID-19 and its complications than the general population (9). However, cancer patients in developing countries with limited resources encounter more serious problems during outbreaks, because the healthcare systems do not prioritize them (13). Cancer patients are also under stress as a result of COVID-19-related conditions, making it difficult for them to visit hospitals and seek treatment (14), which causes anxiety in these patients. Anxiety levels in cancer patients ranged from 15.22 percent (15) to 28.3 percent (16). According to Boy et al. (17) 24 percent of the adult cancer patients experienced anxiety, 14 percent experienced depression, and 69 percent experienced anxiety and depression.

According to a review of the literature, cancer patients used non-pharmacological methods to relieve anxiety and stress. Today, in addition to drug therapy, one of the most common methods for controlling symptoms and strengthening the condition of patients with chronic diseases is the use of CAM (18, 19). CAM refers to knowledge, skills, methods based on theories, beliefs, and experiences indigenous to various cultures that are used to maintain health, prevent, recover from, or treat physical and mental diseases (20). During the COVID-19 outbreak, some types of CAM, particularly nutritional supplements, medicinal herbs, and prayer, were commonly used to prevent COVID-19 and reduce pandemic-related anxiety (21). Cancer patients are motivated to use CAM to cope with pain, insomnia, persistent mental distress and treatment complications (22). Cancer patients in the study of Holmes et al. used 1–18 CAM methods (23). Jazieh et al. investigated the trend of CAM use in cancer patients between 2006 and 2018, finding that 78.9 percent used CAM between 2006 and 2008 and 96.8 percent between 2016 and 2018. The widespread use of CAM among cancer patients indicates significant social and cultural changes in this population (24). Bar-Sela et al. discovered that cancer patients who used CAM six times per week experienced significant reductions in anxiety (25).

The number of cancer survivors is growing (26) and these patients experience ongoing physical symptoms as well as a variety of psychological symptoms, including anxiety and fear of recurrence (16, 27). This group of patients uses CAM to improve and alleviate their physical and psychological symptoms. As there have been few studies in this field, the present study was conducted with the following specific objectives: (a) assessing the psychosomatic symptoms of terminally ill cancer patients based on the EASS and HADS, (b) assessing the frequency of using CAMs, the reasons for using each type of CAMs, and satisfaction with using CAMs, (c) assessing the association between demographic and clinical characteristics and psychosomatic symptoms, (d) assessing the association between using CAMs (Yes/No) and psychosomatic symptoms, and finally (e) as Iran is a religious country and preying is very common in all population, assessing the association between using CAMs without including praying (Yes/No) and psychosomatic symptoms in terminally ill cancer patients.

## Methods and Materials

### Study Design and Setting

This is a cross-sectional study on the relationship between terminally ill cancer patients' use of CAM and their psychosomatic symptoms in Iran. Data was collected from January to August 2021.

### Sampling and Sample Size

The researcher referred to two centers and doctors' offices in Kerman and began sampling. Patients with advanced and terminal cancer (patients with stage 3 to 4 cancer with metastatic stage, cancer patients who can't be cured and aren't responding to treatment, and that the person is likely to die from cancer according to physicin dianosis) were considered for inclusion and incomplete questionnaires were excluded. As we did not find a similar study that assessed the correlation between ESAS, HADS, and CAM usage, we used the Cochran's formula for an infinite population to estimate the sample size (*Z* = 1.96, *d* = 0.07, *n* = 196). According to dropout probability, 250 questionnaires were distributed. Finally, 221 participants completed the questionnaire. The response rate was 89%. Assuming a power of 80% and a *p*-value of 0.05, 211 people would be needed to detect an effect size of 0.28 with G^*^Power version 3.1.9.2.

### Ethical Issue

The ethics committee of Kerman University of Medical Sciences approved the study protocol (IR.KMU.REC.1399.445). Participants signed an informed consent form before beginning the research. The study's objectives, confidentiality, and anonymity were described, and volunteers were given full authority to complete the questionnaire.

### Questionnaire

#### Demographic Information Questionnaire

The demographic and clinical information questionnaire included age, sex, marital status, education level, job, income, living place, type of insurance, type of cancer, duration of cancer, a history of addiction, a history of diabetes, a history of hypertension, a history of cardiovascular disease, a history of other chronic diseases, and a history of hospitalization.

#### Edmonton Symptom Assessment Scale

The Edmonton Symptom Assessment Scale (ESAS) is a 10-item patient-rated symptom visual analog scale developed for use in the symptom assessment of palliative care patients. In the ESAS, patients rate the severity of the following nine symptoms on a 10-cm line: pain, activity, nausea, depression, anxiety, drowsiness, lack of appetite, wellbeing, and shortness of breath. There is an optional tenth symptom, which can be added by the patient. The sum of patient responses to these ten symptoms, in millimeters, is the ESAS distress score. When filling out the questionnaire about cancer symptoms, participants utilized an 11-point scale to score the severity of their symptoms during the last 24 h, with zero being “no” and 10 being “the worst possible” (28). Therefore, the total score of ESAS ranges between 0 and 100. Richardson and Jones used test-retest to assess this instrument's reliability and came up with a reliability coefficient of 0.8 (29). In Iran, Cronbach's alpha for the overall ESAS was 0.88, and the correlation between test-retest was 0.86 in Iranian patients with cancer (30).

#### Hospital Anxiety and Depression Scale

Zigmond and Snaith devised the scale to measure anxiety and depression in a general medical population of patients. This questionnaire is graded based on a four-point scale (31). Kaviani et al. approved the validity and reliability of this questionnaire. Its reliability coefficient was 0.53 for anxiety and 0.59 for depression using the Pearson correlation coefficient (32). Scors for each item range from 0 to 3, with a maximum possible score of 21 for each subscale. A cut-off score of 8 or more for each subscale is recommended for identifying “possible caseness” of anxiety and depression (31). In addition, scores between 8 and 10 indicated mild, 11–14 moderate, and 15–21 severe anxiety or depression (32).

#### Complementary and Alternative Medicine Questionnaire

Complementary and Alternative Medicine Questionnaire Dehghan et al. developed this questionnaire. This questionnaire is about the application of CAM, which includes 10 questions on the use of some types of CAM [herbal medicines, wet cupping, dry cupping, massage, acupuncture, acupressure, homeopathy, relaxation techniques such as yoga, and prayer (the act or practice of praying with God)]. Each one is scored based on a yes/no scale. If the answer was yes, the participants were asked how many times they did it on a 6-point Likert scale (once a year = 1 to every day = 6). Reasons for using CAM were also measured using three options: reducing physical symptoms, reducing anxiety and stress, and others. A yes/no question asked if you should consult your doctor before taking complementary and alternative medicine. To determine the validity, the questionnaire was given to 10 faculty members of Razi School of Nursing and Midwifery in Kerman, and the content validity index of the questionnaire was calculated to be 0.96. Cronbach's alpha coefficient was 0.85 (20).

#### Satisfaction With the Use of Complementary Medicine Questionnaire

Ghaedi et al. developed this questionnaire in Iran. This questionnaire consists of 9 items regarding access to the method, ease of use, harmlessness, non-interference with daily activities, reduction of physical and mental symptoms, non-interference with other treatments, recommendation of the method to others, and affordability (from completely satisfied = 4 to completely dissatisfied = 0). The score range of satisfaction with complementary medicine was between 0 and 36, with a higher score indicating more satisfaction (minimum 9 and maximum 36). The validity of the questionnaire in Ghaedi's et al. studies was obtained using face and content validity, and its internal consistency was obtained to be 0.85 using Cronbach's alpha coefficient (33).

#### Data Collection

The researcher chose medical facilities and offices for sampling, and after consulting with physicians and centers, began sampling in various shifts throughout the morning, evening, and night. The study's objectives and methodology were presented to the participants, and their written consent was acquired. The researcher conducted interviews with individuals who were illiterate and therefore unable to complete the questionnaires.

#### Data Analysis

Descriptive and inferential statistics as well as SPSS 25 were used to analyze the data. Descriptive statistics were used to describe demographic characteristics and mean scores. To determine the relationship between the CAM questionnaire, physical symptoms, anxiety and depression, the Mann–Whitney *U*-test and independent *t*-test were used. To determine the relationship between the psychosomatic symptoms and demographic ans clinical charesteristics, the Mann–Whitney *U*-test, independent *t*-test, Analysis of variance, and Kruskal–Wallis were used. A significance level of 0.05 was considered.

## Results

### Participants' Demographic and Clinical Characteristics

The mean age of the participants was 51.66 ± 13.34 years. The majority of the samples were female, married, educated, and housewives (Table 1). The majority of female patients had breast or ovarian cancer, whereas the majority of male patients had blood cancer, and they were all undergoing chemotherapy. In addition, all participants were at stage 3 or 4 of cancer. The vast majority of participants did not have a history of chronic disease (Table 2).

**Table 1:** Demographic characteristics of the participants and psychosomatic symptoms differences among the participants.

**Variable**	**Frequency** **(valid percent)**	**Physical symptoms**		**Psychological symptoms**	
		**Mean (SD)**	**Statistic test** **(*P*-value)**	**Mean (SD)**	**Statistic test** **(*P*-value)**
Age (yr.)^*^					
≤ 30	16 (7.2)	19.75 (18.50)		6.5 (6.27)	
31–40	29 (13.2)	24.86 (18.46)		6.52 (5.46)	
41–50	58 (26.4)	23.55 (18.33)	*F* = 0.40 (0.81)	6.12 (4.86)	*F* = 0.80 (0.53)
51–60	48 (21.8)	20.90 (17.10)		5.08 (4.21)	
>60	69 (31.4)	21.80 (17.0)		6.68 (5.12)	
**Sex**					
Female	121 (54.8)	23.87 (18.82)	*Z* = −1.0 (0.32)	6.76 (4.99)	*t* = −2.08 (0.04)
Male	100 (45.2)	20.29 (15.81)		5.37 (4.91)	
**Marital status**					
Single	24 (10.9)	16.58 (12.53)		4.67 (4.90)	
Married	187 (84.6)	22.34 (18.02)	*H* = 8.20 (0.02)	6.28 (5.01)	*F* = 1.24 (0.29)
Divorced/widow(er)	10 (4.5)	34.20 (13.93)		6.90 (4.72)	
Education level^*^					
Uneducated	43 (19.5)	21.53 (17.30)		6.30 (4.62)	
Elementary school	48 (21.8)	21.31 (16.39)		5.73 (4.56)	
Middle/high school	39 (17.7)	22.95 (17.21)	*F* = 0.31 (0.87)	6.46 (6.25)	*H* = 3.34 (0.50)
Diploma	60 (27.4)	24.20 (19.48)		6.93 (5.29)	
Academic	30 (13.6)	20.70 (16.86)		4.70 (3.34)	
Job^*^					
Employed	10 (4.5)	19.70 (17.74)		2.90 (2.73)	
Self-employed	64 (29.1)	18.14 (15.59)		4.95 (4.44)	
Housewife	92 (41.8)	24.34 (19.23)	*H* = 6.11 (0.19)	7.13 (5.14)	*H* = 13.86 (0.008)
Retired	29 (13.2)	25.10 (16.28)		7.31 (5.68)	
Unemployed	25 (11.4)	23.68 (16.34)		5.64 (4.58)	
Income (Million Tomans)^*^					
<1	101 (45.9)	24.48 (19.06)		6.92 (5.20)	
1–2	39 (17.7)	20.77 (18.85)	*H* = 1.59 (0.45)	5.64 (4.96)	*F* = 2.22 (0.11)
>2	80 (36.4)	20.42 (14.56)		5.45 (4.62)	
**Living place**					
Urban	190 (86.0)	22.39 (18.02)	*t* = 0.29 (0.77)	6.10 (5.03)	*t* = −0.23 (0.82)
Rural	31 (14.0)	21.39 (14.80)		6.32 (4.82)	
Insurance^*^					
Yes	195 (88.6)	22.18 (17.67)	*t* = −0.55 (0.58)	6.16 (5.07)	*t* = −0.001 (0.99)
No	25 (11.4)	24.16 (16.84)		6.16 (4.36)	

* *Missing value, SD, Standard Deviation; t, Independent t-test; Z, Mann–Whitney U; F, Analysis of variance; H, Kruskal–Wallis H; One Dollar was nearly 25,000 Tomans at the time of sampling*.

**Table 2:** Clinical characteristics of the participants and psychosomatic symptoms differences among the participants.

**Variable**	**Frequency** **(valid percent)**	**ESAS score**		**HADS score**	
		**Mean (SD)**	**Statistic test (*P*-value)**	**Mean (SD)**	**Statistic test (*P*-value)**
**Type of cancer**					
Breast	75 (33.9)	24.48 (17.80)	*F* = 0.65 (0.66)	6.69 (5.27)	*F* = 0.98 (0.43)
Blood	58 (26.2)	19.72 (15.79)		5.51 (4.58)	
Ovary	23 (10.4)	24.22 (18.96)		6.61 (6.07)	
Gastrointestinal	26 (11.8)	21.46 (19.34)		4.77 (3.56)	
Lung	24 (10.9)	19.71 (15.79)		7.08 (4.72)	
Others	15 (6.8)	23.27 (21.14)		5.80 (5.76)	
<3	49 (22.2)	20.51 (16.79)	*F* = 1.28 (0.28)	5.43 (5.15)	*F* = 2.64 (0.04)
3–6	67 (30.3)	19.34 (16.28)		5.10 (4.53)	
7–12	47 (21.3)	24.51 (18.33)		7.40 (5.07)	
13–36	35 (15.8)	24.20 (17.12)		6.11 (4.38)	
>36	23 (10.4)	26.83 (21.26)		8.04 (5.90)	
**History of opium addiction**					
Yes	99 (44.8)	22.24 (15.66)	*Z* = −0.88 (0.38)	6.07 (4.93)	*t* = −0.16 (0.87)
No	122 (55.2)	22.25 (19.05)		6.18 (5.06)	
**History of diabetes**					
Yes	26 (11.8)	24.04 (19.19)	*t* = −0.55 (0.58)	5.85 (4.34)	*t* = 0.31 (0.76)
No	195 (88.2)	22.01 (17.39)		6.17 (5.08)	
**History of hypertension**					
Yes	36 (16.3)	21.50 (17.45)	*t* = 0.28 (0.78)	6.61 (5.29)	*t* = −0.63 (0.53)
No	185 (83.7)	22.39 (17.64)		6.04 (4.94)	
**History of cardiovascular disease**					
Yes	17 (7.7)	28.35 (15.96)	*t* = −1.50 (0.14)	7.12 (4.72)	*t* = 0.85 (0.40)
No	204 (92.3)	21.74 (17.64)		6.05 (5.02)	
**History of other chronic disease**					
Yes	17 (7.7)	28.76 (16.64)	*t* = −1.60 (0.11)	5.59 (3.02)	*Z* = −0.24 (0.81)
No	204 (92.3)	21.71 (17.58)		6.18 (5.12)	
History of hospitalization^*^					
Yes	149 (69.0)	24.34 (18.21)	*Z* = −2.34 (0.02)	6.52 (5.08)	*t* = 1.52 (0.13)
No	67 (31.0)	18.21 (15.87)		5.40 (4.81)	

* *Missing value, ESAS, Edmonton symptom assessment scale; HADS, Hospital anxiety and depression scale; SD, Standard deviation; t, Independent t-test; F, ANOVA; H, Kruskal–Wallis H; Z, Mann–Whitney U*.

### Psychosomatic Symptoms of Terminally Ill Cancer Patients

The mean score for the physical symptoms of the participants according to ESAS was 22.25 ± 17.57, which was less than the midpoint of the scale, i.e., 50. The mean and standard deviation of the following nine symptoms: pain, activity, nausea, depression, anxiety, drowsiness, lack of appetite, wellbeing, and shortness were 2.47 (2.98), 3.28 (3.20), 1.63 (2.65), 1.42 (2.45), 1.90 (2.61), 2.73 (2.75), 3.12 (3.04), 3.36 (2.82), and 1.21 (2.26), respectively.

The mean score for the psychological symptoms of the participants was 6.13 ± 4.99. The mean score for the anxiety subscale was 2.61 ± 2.46. Only 2.7% (*n* = 6) and 0.9% (*n* = 2) of the participants had mild and moderate anxiety, respectively, and the other participants' anxiety levels were normal. The mean score for the depression subscale was 3.52 ± 3.18. Only 7.7% (*n* = 17) and 4.1% (*n* = 9) of the participants had mild and moderate depression, respectively, and the other participants' depression levels were normal.

### CAM Uses and Satisfaction in Terminally Ill Cancer Patients

Overall, 87.3 percent of participants reported using at least one type of CAM in the previous year. Regardless of prayer, 42.1 percent of participants reported using at least one type of CAM in the previous year. Additionally, 48.4 percent (*n* = 107) of individuals used only one form of CAM, 29.9 percent (*n* = 66) used two types of CAMs, 5.4 percent (*n* = 12) used three types of CAMs, and 3.6 percent (*n* = 8) used four to five types of CAMs in the last year. 83.7% of participants reported having used prayer, 35.8% reported using medicinal herbs, 9.5% reported using massage, 3.6% reported using dietary supplements, 3.2% reported using wet cupping, 2.7% reported using relaxation and meditation, 2.4% reported using dry cupping, and 0.5% reported using acupuncture (Table 3). In all the methods of CAM, the common reason for using CAM was to reduce stress and anxiety resulting from cancer and its treatment. 62.5, 42.9, and 20% of participants, respectively, consulted a physician regarding the usage of dietary supplements, wet cupping, and dry cupping. None of the individuals sought medical advice before using medicinal plants. The mean score of satisfaction with CAM use was 11.97 ± 13.24 (Min = 0 and Max = 36), which was less than the scale midpoint of 18.

**Table 3:** The use of CAMs and the reasons for using each type of CAMs in terminally ill cancer patients.

**Variable**	**Frequency of the** **users (%)**	**95% confidence interval of percentage (%)**	**Reasons for using the CAM methods [*****n*** (%)^*^]		
			**Reducing physical complications of the cancer and its treatment**	**Reducing stress and anxiety resulting from cancer and its treatment**	**Others**
Medicinal herbs	76 (35.8)	57.5–70.3	10 (13.2)	52 (68.4)	14 (18.4)
Dry cupping	5 (2.4)	0.5–4.7	–	4 (80)	1 (21)
Wet cupping	7 (3.2)	0.9–5.5	–	5 (83.3)	1 (16.7)
Massage	21 (9.5)	5.9–13.6	1 (4.8)	18 (85.7)	2 (9.5)
Dietary supplements	8 (3.6)	1.4–6.4	–	4 (57.1)	3 (42.9)
Acupressure	1 (0.5)	0–1.4	–	1 (100)	–
Relaxation and meditation	6 (2.7)	0.9–5.0	2 (33.3)	3 (50.0)	1 (16.7)
Prayer	185 (83.7)	78.7–88.7	41 (23.7)	87 (50.3)	45 (26.0)

* *Valid percent, CAM, complementary and alternative medicines; Others, strengthening the immune system, decreasing fatigue*.

### The Association Between Psychosomatic Symptoms, CAMs Use, and Other Study Variables in Terminally Ill Cancer Patients

There were no significant differences in physical and psychological symptoms between the CAM-users and non-CAM users. When excluding praying, the CAM users had significantly higher scores in activity, nausea, drowsiness, and wellbeing than non-CAM users. In addition, the CAM users had a significantly lower score for shortness of breath and other symptoms than non-CAM users (Table 4). Among other study variables, only females and patients with a longer history of cancer had significantly higher psychological symptoms than others. In addition, employed participants had fewer psychological symptoms than non-employees (Tables 1, 2).

**Table 4:** The psychosomatic symptoms of terminally ill cancer patients and its association with CAM usage.

	**Variable**	**CAM user with prayer**		**Statistic test** **(*P*-value)**	**CAM user without prayer**		**Statistic test** **(*P*-value)**
		**Yes** **(Mean/SD)**	**No** **(Mean/SD)**		**Yes** **(Mean/SD)**	**No** **(Mean/SD)**	
Edmonton symptom assessment scale	Pain	2.49 (3.06)	2.28 (2.43)	*Z* = −0.30 (0.76)	2.52 (3.27)	2.43 (2.77)	*Z* = −0.91 (0.36)
	Activity	3.44 (3.27)	2.14 (2.32)	*Z* = −1.71 (0.09)	4.16 (3.66)	2.64 (2.65)	*Z* = −2.44 (0.02)
	Nausea	1.69 (2.73)	1.18 (2.0)	*Z* = −0.34 (0.73)	2.30 (3.17)	1.14 (2.08)	*Z* = −2.19 (0.03)
	Depression	1.40 (2.48)	1.54 (2.30)	*Z* = −1.46 (0.14)	1.74 (2.87)	1.20 (2.08)	*Z* = −0.08 (0.94)
	Anxiety	1.93 (2.64)	1.71 (2.39)	*Z* = −0.02 (0.98)	2.21 (3.02)	1.68 (2.26)	*Z* = −0.57 (0.57)
	Drowsiness	2.85 ± 2.78	1.89 (2.42)	*Z* = −1.86 (0.06)	3.30 (3.0)	2.31 (2.49)	*Z* = −2.49 (0.01)
	Lack of appetite	3.28 (3.08)	2.14 (2.63)	*Z* = −1.91 (0.06)	3.63 (3.38)	2.77 (2.73)	*Z* = −1.55 (0.12)
	Wellbeing	3.41 (2.88)	2.96 (2.44)	*Z* = −0.54 (0.59)	3.92 (3.01)	2.95 (2.62)	*Z* = −2.26 (0.02)
	Shortness of breath	1.20 (2.24)	1.25 (2.42)	*Z* = −0.04 (0.96)	0.79 (1.73)	1.51 (2.54)	*Z* = −2.19 (0.03)
	Other symptoms	1.16 (2.29)	1.61 (2.66)	*Z* = −1.07 (0.28)	0.42 (1.23)	1.79 (2.76)	*Z* = −3.97 (<0.001)
	Total score	22.76 (17.56)	18.71 (17.57)	*t* = −1.14 (0.26)	24.76 (19.51)	20.42 (15.85)	*Z* = −1.28 (0.20)
Hospital anxietyand depressionscale	Anxiety	2.52 (2.39)	3.21 (2.88)	*Z* = −1.14 (0.26)	2.53 (2.25)	2.67 (2.61)	*Z* = −0.01 (0.99)
	Depression	3.55 (3.23)	3.29 (2.90)	*Z* = −0.32 (0.75)	3.92 (3.36)	3.23 (3.03)	*Z* = −1.59 (0.11)
	Total score	6.08 (4.97)	6.50 (5.25)	*t* = 0.42 (0.68)	6.45 (4.98)	5.90 (5.0)	*t* = −0.81 (0.42)

*SD, Standard deviation; t, Independent t test; Z, Mann–Whitney U*.

## Discussion

The purpose of this study was to examine the relationship between psychosomatic symptoms and the usage of CAMs in terminally ill cancer patients. Only 3.6 percent of the individuals in this study reported having mild to moderate anxiety. Only 11.8% of subjects reported having mild to moderate depression. In addition, the level of physical symptoms was low, according to ESAS. In line with the results of the present study, Walker et al. discovered that depression prevalence was between 5 and 16% in outpatients but was 7–49% in patients under palliative care (34). Contrary to the results of the present study, other studies have reported higher levels of anxiety and stress. In a study by Kolva et al. moderately elevated anxiety symptoms were found in 18.6% of terminally ill cancer patients, and 12.4% had clinically significant anxiety symptoms (35). In a study by Salvo et al., also fifty-five percent of patients with advanced cancer reported at least mild symptoms of depression and 65% reported at least mild anxiety (36). Dehghan et al. found that 61.4 percent of individuals reported moderate to severe anxiety on the Corona Diseas Anxiety Scale (CDAS) and 38 percent had moderate to severe anxiety on the subscale of physical symptoms (16). Ayubi et al. demonstrated that the prevalence of depression and anxiety in cancer patients could increase dramatically during the COVID-19 outbreak (37). Studies indicated a decreased prevalence of anxiety and depression before the COVID-19 outbreak. According to Dehghan et al. 28.3 percent of cancer patients experienced moderate to severe anxiety (16). According to Amatory et al. 15.22 percent of cancer adults and adolescents had anxiety (15). Boy et al. discovered that 24% of adult cancer patients experienced anxiety, 14% experienced depression, and 69% experienced both anxiety and depression (17). Oncologic patients are an extremely sensitive population, and cancer is frequently viewed as a life-threatening condition associated with a high risk of mortality, which may explain why patients are fearful of problems related to crises in addition to the disease's outcomes. COVID-19 may cause concern for cancer patients due to its effect on cancer treatment, recurrence, and progression. This may predispose these patients to anxiety and depression symptoms (37). However, we think one of the most important reasons for the lower level of psychosomatic symptoms in our sample was the unawareness of the majority of the participants about their cancer diagnosis. In our study setting, there was a huge request from patients' families that their patients be kept unaware of their diagnosis, and researchers should gather data in such a way that terminally ill cancer patients are kept unaware of their diagnosis.

Several factors, however, can influence the reports. Sigorsk et al. demonstrated that anxiety and fear varied significantly by tumor type. Patients with breast cancer reported the highest levels of anxiety, whereas those with lung cancer reported the lowest levels (38). Also, Wasteson et al. have previously commented that the lack of a consistent measurement and definition of depression in people with cancer makes comparisons of prevalence estimates problematic and that the range of experience and training of interviewers employed in prevalence studies adds to this problem (39). Our sampling was performed under coronavirus conditions, and these conditions may have found patients to be deviating from the main path of thought. However, in order to confirm the results of the present study, future studies should pay attention to other factors affecting anxiety and depression, such as personal, family, and social conditions.

The present study showed that, other than prayer, 87.3% used at least one type of CAM in the past year. 42.1% of participants used at least one type of CAM in the past year. Most of the participants used prayer (83.7%) and medicinal herbs (35.8%). Consistent with the results of the present study, John et al. showed that 36.4% of cancer survivors used at least one CAM method other than vitamins in the past year, and 38.9% used only vitamins and minerals (40). Albabtain et al. showed that 81.1% of Saudi women with breast cancer used CAMs, with spiritual therapy being the most popular (70.5%) (41). Contrary to the results of the present study, some other studies have found that other CAM methods are more often used. Holmes et al. showed that there were 1 to 18 treatments, with massage (82%) being the most common, followed by acupuncture (64%), and the use of vitamins and minerals (64%) (23). Qureshi et al. showed that 75% of cancer survivors used CAMs, with 21% using mental complementary therapies and up to 11% using energy-based complementary therapies (42).

Some studies have discussed the role of complementary and alternative medicine in the management of cancer patients (43, 44). These results demonstrate that cultures and communities have an effect on the quantity and kind of CAM use. Given Iran's religious tradition as an Islamic country, the high rate of prayer practiced in this study was expected. The usage of medicinal plants came in second. Consistent with the present study's results, Er et al. discovered that 90% of patients utilized herbal medicines (45). Additionally, Tarhan et al. demonstrated that the most frequently used CAM method in cancer patients was plant products (36.3 percent) (46), which is quite similar to the current study. Herbal medicines, on the other hand, are advised for the treatment and alleviation of patients' symptoms (47). According to Sheikhrabori et al. (48), the most important reasons for using herbal medicines were their ease of use, their safety, contentment with symptom treatment, and lack of concern about drug interactions. Additionally, Iranians appear to be more receptive to and trusting of herbal medicines.

The current study demonstrated that the primary motive for using CAM was to alleviate stress and anxiety caused by cancer and its treatment. Consistent with the findings of this study, Hann et al. discovered that 64% of breast cancer survivors used CAMs to manage disease-related stress and anxiety (49). Sela et al. demonstrated that cancer patients who used CAM six times a week experienced significant reductions in anxiety (25). Salek et al. discovered that using crocin during chemotherapy in patients with breast cancer has ameliorated anxiety and depression (50).

Terminally ill cancer patients face increased stress and decreased immunity. As a result, they may use CAM more frequently to alleviate stress and enhance their immune systems (51). However, alternative indications for the use of CAMs include cancer treatment and symptom management, such as pain control and appetite enhancement (24). Albabtain et al. discovered that many women used CAM to improve their physical and emotional wellbeing, with the primary cause being improved immune system function (41). According to Al-Naggar et al. 65.5 percent of cancer patients claimed that CAM was beneficial to them, 92 percent reported no adverse effects, and 80 percent expressed satisfaction with CAM. 19.5 percent used CAM to alleviate pain, and 16.5 percent used it to alleviate symptoms (52). Due to the increased interest in CAM, healthcare practitioners should be educated about the most commonly used CAM methods for cancer patients and assist them in selecting and using the appropriate method.

Another significant result from this study was the frequency with which cancer patients sought advice from physicians regarding the use of CAM. The current study discovered that the majority of recommendations focused on the usage of dietary supplements (62.5 percent). Although the present study found a significant prevalence of medicinal plant use, none of the participants visited a physician before utilizing medicinal plants. While studies have not specified the extent to which patients consult physicians, one of the primary reasons for the use of herbal medicines has been consumers' lack of awareness of herbal medicine's toxicity (53). However, several studies have connected the use of herbal medicines to cardiac arrhythmias (54) and vascular problems (55). Another issue that has attracted people's interest is the availability of herbal medicines. Although no adverse events were identified in this study as a result of cancer patients' using CAM, it is vital to adopt measures to encourage patients to report their use of herbal medicines and to pay closer attention to probable negative effects. Additionally, the critical issue is to convince patients to consult with doctors, which requires cultivating a culture and educating patients in this regard. A study conducted between 2006 and 2008 as well as between 2016 and 2018 found that disclosure of CAM use had not changed significantly over time and remained low (31.6–35.7 percent), indicating that additional research and interventions are needed to identify the causes and increase disclosure of information to physicians by cancer patients (24). Furthermore, the absence of management advice and data on the quantity and quality of CAM-related recommendations in clinical practice guidelines (CPGs) for treatment or management demonstrates a significant gap in the necessary guidance for physicians and clinical researchers (56).

In the current study, there was no significant difference in physical or psychological symptoms between CAM and non-CAM users. In addition, when excluding prayer, CAM users were worse off in activity, nausea, drowsiness, and wellbeing than non-CAM users, but they were better than non-CAM users in the symptoms of shortness of breath and other symptoms. Consistent with the findings of this study, Saini et al. found that CAM users scored worse on physical wellbeing than non-CAM users in Italy, and that there was no significant difference in anxiety, depression, and coping methods between the two groups (57). In contrast to the current study's results, Albabtain et al. (41) showed that Saudi women undergoing breast cancer treatment used a much larger percentage of CAM than those who were not treated for cancer. There was a statistically significant difference in quality of life between CAM and non-CAM users. Additionally, the research population's usage of CAM was influenced by a variety of characteristics, including employment, monthly income, and ongoing cancer treatment (41). In Korea, Jang et al. discovered that CAM use is connected with lower levels of anxiety and depression than non-CAM use, and that greater levels of education, income, fewer doctor consultations, and an advanced stage of cancer are all substantially correlated with CAM use (58). According to Dastgheib et al. (59), 89.9 percent of patients with skin conditions such as acne and alopecia used some form of CAM. This result demonstrates that the kind and course of the disease are significant factors influencing the use and effectiveness of CAM. Additionally, Peltzer and Pengpid (60) demonstrated that TCAM was related to characteristics such as poor health, depression, and chronic illness or disability. These findings underscore the importance of conducting additional research on the impact of CAM on physical and mental symptoms in CAM and non-CAM users.

Although use of CAM is widespread, the underlying reasons patients choose CAM are not clearly understood. Several explanatory models have been suggested, including the desire for personal control, compatibility with holistic beliefs, and dissatisfaction with conventional care. Patients use CAM when it is consistent with their worldview and conventional care is not relieving their symptoms (61). Based on the cancer patients' statements was developed a model representing the viewpoints and thought patterns of CAM users as contrasted with those patients who did not use CAM including a view of CAM as safe and holistic coupled with a view of conventional medicine as an aggressive and isolated treatment; concern about side effects; a belief in the potential efficacy of CAM despite the lack of evidence; and a need to gain a sense of control. Multiple ideas woven together led patients toward CAM use. An understanding of patients' thought processes may aid health care professionals in initiating a dialogue about decision-making and potential side effects of using CAM (62).

However, people are attempting to improve their health with CAM. A review of the literature revealed that patients' perceptions of CAM use might also influence CAM utilization and engagement. In China, cancer patients lacked knowledge of the benefits of participation in clinical trials of complementary and alternative medicine (CTCAM). However, a substantial number of cancer patients expressed an interest in participating in CTCAM (63). To have a better understanding of this issue, future research should focus on individual and social aspects to ascertain the reasons for acceptance, use, and type of effect of CAM on cancer patients. The current study delves deeper into CAM and provides health practitioners with additional information about CAM use and its effects on cancer patients.

## Limitations

Our study has limitations. Due to the cross-sectional nature of this study, the cause-and-effect relationship is unknown; more interventional and longitudinal studies are recommended in this area. In addition, as the study population was at end-stage phase and almost had the same comorbidities and performance status, and to reduce the length of the questionnaire, we did not ask them about these variables. Further studies with larger sample size and case control design are recommended as the frequency of CAM users and non-CAM users were not the same among our terminally ill cancer patients and non-parametric tests were used in the majority of the cases. Given that the study population consisted of cancer patients in southeastern Iran, caution should be applied when generalizing the results.

## Conclusion

This study discovered that cancer patients exhibited low levels of psychosomatic symptoms. Additionally, the primary rationale for employing CAM has been to alleviate the stress and anxiety associated with cancer and treat it. Further studies are needed to identify the factors that affect knowledge and motivation to use CAM in cancer patients. Another significant conclusion from this study was the absence of differences in physical and psychological symptoms between CAM and non-CAM users. Future research should look into how CAM can help people with cancer and how it can hurt them.

## Data Availability Statement

The raw data supporting the conclusions of this article will be made available by the authors, without undue reservation.

## Ethics Statement

The studies involving human participants were reviewed and approved by Kerman University of Medical Sciences. The patients/participants provided their written informed consent to participate in this study.

## Author Contributions

MD, FH, and MZ develop the study idea and protocol. MD supervised the study sampling. FH did the sampling. MD analyzed the data. MD and MZ wrote the first draft of the manuscript. All authors read and confirmed the final version of the manuscript.

## Conflict of Interest

The authors declare that the research was conducted in the absence of any commercial or financial relationships that could be construed as a potential conflict of interest.

## Publisher's Note

All claims expressed in this article are solely those of the authors and do not necessarily represent those of their affiliated organizations, or those of the publisher, the editors and the reviewers. Any product that may be evaluated in this article, or claim that may be made by its manufacturer, is not guaranteed or endorsed by the publisher.
